# Temperature sensitive liposomes combined with thermal ablation: Effects of duration and timing of heating in mathematical models and *in vivo*

**DOI:** 10.1371/journal.pone.0179131

**Published:** 2017-06-12

**Authors:** Christian Rossmann, M. A. McCrackin, Kent E. Armeson, Dieter Haemmerich

**Affiliations:** 1 Department of Pediatrics, Medical Univ. of South Carolina, Charleston, South Carolina, United States of America; 2 Department of Comparative Medicine, Medical Univ. of South Carolina, Charleston, South Carolina, United States of America; 3 Ralph H. Johnson Veterans Affairs Medical Center, Charleston, South Carolina, United States of America; 4 Hollings Cancer Center, Medical Univ. of South Carolina, Charleston, South Carolina, United States of America; 5 Department of Bioengineering, Clemson Univ., Clemson, South Carolina, United States of America; Case Western Reserve University, UNITED STATES

## Abstract

**Background:**

Temperature sensitive liposomes (TSL) are nanoparticles that rapidly release the contained drug at hyperthermic temperatures, typically above ~40°C. TSL have been combined with various heating modalities, but there is no consensus on required hyperthermia duration or ideal timing of heating relative to TSL administration. The goal of this study was to determine changes in drug uptake when heating duration and timing are varied when combining TSL with radiofrequency ablation (RF) heating.

**Methods:**

We used computer models to simulate both RF tissue heating and TSL drug delivery, to calculate spatial drug concentration maps. We simulated heating for 5, 12 and 30 min for a single RF electrode, as well as three sequential 12 min ablations for 3 electrodes placed in a triangular array. To support simulation results, we performed porcine *in vivo* studies in normal liver, where TSL filled with doxorubicin (TSL-Dox) at a dose of 30 mg was infused over 30 min. Following infusion, RF heating was performed in separate liver locations for either 5 min (n = 2) or 12 min (n = 2). After ablation, the animal was euthanized, and liver extracted and frozen. Liver samples were cut orthogonal to the electrode axis, and fluorescence imaging was used to visualize tissue doxorubicin distribution.

**Results:**

Both *in vivo* studies and computer models demonstrate a ring-shaped drug deposition within ~1 cm of the visibly coagulated tissue. Drug uptake directly correlated with heating duration. In computer simulations, drug concentration increased by a factor of 2.2x and 4.3x when heating duration was extended from 5 to either 12, or 30 minutes, respectively. *In vivo*, drug concentration was by a factor of 2.4x higher at 12 vs 5 min heating duration (7.1 μg/g to 3.0 μg/g). The computer models suggest that heating should be timed to maximize area under the curve of systemic plasma concentration of encapsulated drug.

**Conclusions:**

Both computer models and *in vivo* study demonstrate that tissue drug uptake directly correlates with heating duration for TSL based delivery. Computational models were able to predict the spatial drug delivery profile, and may serve as a valuable tool in understanding and optimizing drug delivery systems.

## Introduction

Temperature sensitive liposomes (TSL) are liposomal drug carriers which release the contained drug in response to temperatures above ~40°C. Thus, TSL facilitate targeted drug delivery when combined with localized hyperthermia, which can be mediated for example by focused ultrasound or radiofrequency (RF) ablation mediated heating [1, 2]. While long-circulating stealth liposomes as currently in clinical use (e.g. Doxil^®^) passively target the tumor via enhanced permeability and retention (EPR) effect [3, 4], the more recent TSL formulations are based on intravascular triggered release mechanism [5–7]. In this type of delivery strategy, drug is released while the TSL in plasma traverse the tumor vasculature. This strategy requires very rapidly releasing TSL formulations (within seconds) to be most effective [5].

Here, we will specifically focus on TSL filled with doxorubicin (TSL-Dox), and use radiofrequency (RF) ablation as localized heating modality. RF ablation is a clinical cancer therapy used for a variety of solid tumors, and is widely used for liver cancer [8, 9]. Under imaging guidance, a RF electrode is directly inserted into the tumor. Upon application of RF energy to the electrode, heat is generated in the surrounding tissue (up to ~ 100°C), resulting in coagulation necrosis [10, 11]. The combination of RF ablation with TSL-Dox may reduce local recurrence in the margin of the ablation zone where cell kill is not complete (40–50°C) by targeted drug delivery to this region [1, 12–14].

Due to the complex interplay between tissue heating, drug pharmacokinetics and TSL properties, it is not obvious how different heating strategies affect the amount of drug being delivered to the target site for this combination therapy. For example, there is no agreement on optimal timing of heating (i.e. before, during or after TSL administration). Similarly the required heating duration is not known (2–60 minutes have been employed by prior studies) [2, 6, 7, 15–20], and studies investigating impact of heating duration are lacking. Computational models provide an elegant approach to understand the drug delivery kinetics and to optimize delivery strategies. The purpose of this study was to quantify the effect of timing and duration of heating on amount of drug being delivered to the target site, both via computational models and *in vivo* studies.

## Methods

### Computer models

#### Key equations and assumptions

The computer models consisted of two separate biophysics models, both simulated in 3D. The first model was a heat transfer model that simulated tissue heating, including temperature dependent changes in perfusion. The second model simulated drug delivery, including drug release from temperature sensitive liposomes (TSL), transvascular transport of released drug, and cellular drug uptake. The local tissue temperature and perfusion served as spatially and temporally varying input parameters to the drug delivery model, where the local temperature determined release rate of the drug from TSL within plasma.

**Fig 1:**
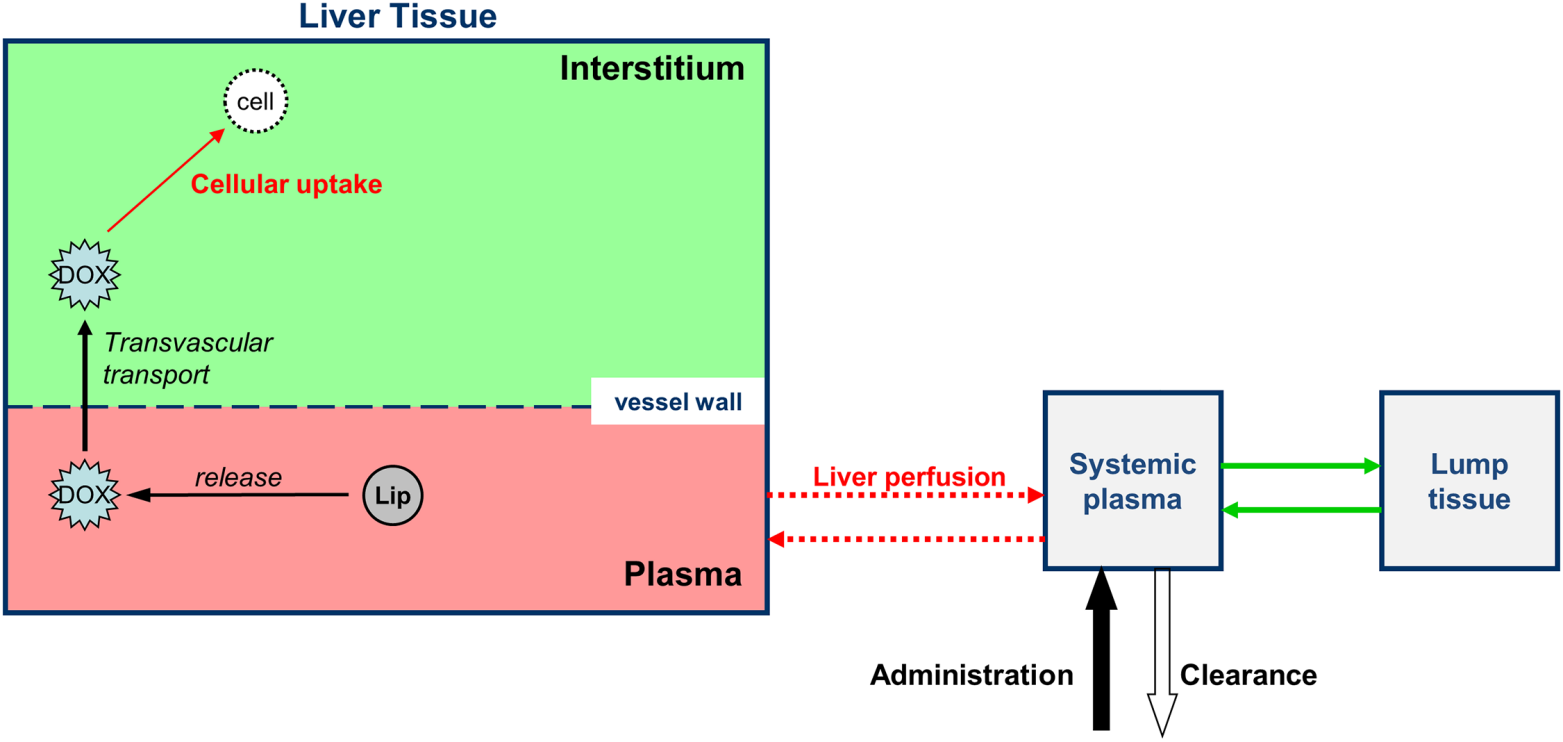
Drug delivery model overview. Intravascular temperature-dependent release of Dox from TSL, uptake by interstitium (EES), and cell uptake are simulated. Local temperature and perfusion are fed into the drug delivery model from the heat transfer model.

The following assumptions have been made:

- Biophysical tissue parameters are assumed uniform, i.e. not spatially varying.
- Following tissue properties are assumed temperature dependent: perfusion, thermal conductivity, specific heat, and electric conductivity.
- Drug transport between plasma and interstitial space was modeled as diffusion process. While convective transport is not explicitly modeled, such transport is assumed to be included in the diffusive transport model.
- Transport of liposomes between plasma and interstitial space is neglected, as a prior study demonstrated it to be negligible within the time frame of ~30 min examined here [5].
- Uptake of liposomes by macrophages and subsequent release of doxorubicin (Dox) is not considered. Due to the comparably slow rate of this transport mechanism compared to TSL based delivery, we do not expect it to have a significant impact.
- Doxorubicin binds extensively (∼70%) to plasma proteins. However, due to the rapid dynamics of drug release from TSL and tissue uptake (within seconds), we assume that plasma protein binding does not significantly affect transvascular transport. Qualitative observations of similar fluorescence intra- and extra-vascularly from prior intravital studies support this assumption [6].
- Cellular doxorubicin uptake by liver cells is based on a prior study and considers bound and unbound drug [21].
- The total tumor tissue drug concentration was calculated by weighted averaging of concentrations in interstitium (unencapsulated and liposomal) and cells, considering the volume fraction of each compartment.
- Systemic body and plasma compartments: The systemic plasma compartment concentration considered inflow and outflow from liver, as well as clearance, and uptake by body tissue. All body tissues except liver were lumped together into one compartment (Fig 1), and transport processes to and from systemic tissues were described by rate constants. No specific tissue types, and no separation of systemic tissues into EES and cellular compartments were considered.
- We assumed that TSL mix perfectly within systemic plasma during infusion.

Key Equations:

Tissue heating was modeled by the following heat transfer equation, where variables are: *T*: temporally and spatially varying tissue temperature; *ρ*: tissue density; *c*: tissue specific heat; *k*: tissue thermal conductivity. *Q*_rf_: radiofrequency energy deposited in tissue; *Q*_p_: heat carried away by perfusion:

$$\rho c\frac{\partial T}{\partial t}=k\nabla^{2}T+Q_{\text{rf}}-Q_{\text{P}}$$

The plasma concentration of unencapsulated drug was modeled as:

$$\frac{dc_{p}{}^{T}}{dt}=-\frac{1}{v_{p}{}^{T}}PS\cdot (c_{p}{}^{T}-c_{e}{}^{T})-F_{pv}{}^{T}c_{p}{}^{T}+F_{pv}{}^{T}c_{p}{}^{B}+c_{p\_TSL}R_{R}$$

This equation describes the rate of plasma concentration change of unencapsulated drug, and is calculated individually at each tissue location (i.e. all variables are spatially dependent). The first right-hand term describes diffusion transport between plasma and interstitium. The second and third right-hand terms describe unencapsulated drug entering and exiting the vasculature of a particular tissue segment, considering local plasma perfusion rate *F*_pv_^T^. The final term describes intravascular drug release from TSL at rate *R*_R_.

Once drug has been transported from the vasculature (plasma) into the interstitium, the drug is taken up by cells. Within cells, bound (*c*_i,b_) and unbound drug (*c*_i,u_) concentrations are modeled by the following two equations:

$\frac{dc_{i,u}{}^{T}}{dt}=k_{1}c_{e}{}^{T}-k_{2}c_{i,u}{}^{T}-k_{3}c_{i,u}{}^{T}$ Dox concentration in liver cells (unbound)

$\frac{dc_{i,b}{}^{T}}{dt}=k_{3}c_{i,u}{}^{T}$ Dox concentration in liver cells (bound)

The constants *k*_1_, *k*_2_, *k*_3_ represent rate constants describing transport between interstitial space (EES), and cellular compartments for bound and unbound drug.

#### Computer model of radiofrequency heating

Similar to prior studies, we coupled a heat-transfer model simulating tissue heating with a drug delivery model, and simulated these in 3-D [1, 2]. In the heat-transfer model, the temporally and spatially varying tissue temperature profile resulting from RF heating was calculated by solving Pennes’ Bioheat equation [22]. We considered spatially and time varying perfusion based on thermal dose dependent perfusion change [23]; this perfusion change was considered both for RF tissue heating and drug delivery models. RF model details are described in the Supporting Information (S1 File).

#### Computational drug delivery model

The tissue temperature and perfusion from the RF heating model were fed into the drug delivery model to calculate intravascular release from TSL-Dox, transport from plasma into tissue interstitium (extracellular-extravascular space (EES)), and uptake by liver cells (Fig 1), similar to prior studies [1, 2].

The three tissue compartments (plasma, EES, cells) were considered spatially varying, i.e. each location within the target region was associated with individual drug concentrations for each compartment. In addition, a systemic lump tissue compartment (representing all body tissues except target tissue), and a systemic plasma compartment were considered. TSL-Dox was assumed administered over 30 min at a dose of 0.6 mg/kg, with volume of distribution equal to the plasma volume. Pharmacokinetics (PK) of Dox after release was based on prior studies [24], as were PK and release kinetics of TSL-Dox [1, 14]. Note that we considered uptake kinetics of normal liver cells [21] rather than tumor cells as in prior studies [1], to allow direct comparison to the corroborating *in vivo* studies performed in normal porcine liver. Detailed equations and parameter values of the computational models are listed in the Supporting Information (S1 File).

Two computer model geometries were developed to simulate a single needle electrode (model 1), or three needle electrodes arranged in a triangular cluster, 2 cm apart (model 2). Model 1 was employed to simulate RF heating for 5, 12 and 30 minutes. In addition, a 12-minute ablation (the clinically used duration for this type of electrode) was simulated starting either immediately, 60 min, or 120 min after administration of TSL-Dox. In Model 2, three sequential 12-minute ablations were simulated, emulating clinical practice where multiple sequential ablations are employed to cover a large tumor.

### In vivo studies

Since there is no adequate large animal tumor model, we used a clinical RF ablation device that is not feasible for small animal use and performed studies in normal porcine liver. All animal studies were approved by the MUSC Institutional Animal Care and Use Committee (IACUC). A female Yorkshire pig (47.6 kg weight) was pre-medicated with methylprednisolone (SC, 80 mg) the day before the procedure and with hydrocortisone (IV, 100 mg) just prior to TSL-Dox infusion. Anesthetic premedication included ketamine (SC, 25 mg/kg), acepromazine (SC, 1.5–2.2 mg/kg), and atropine (SC, 0.04 mg/kg). Isoflurane in oxygen was used for mask induction (4–5%) and maintenance (1.5–3%) after intubation and placement on a ventilator. Continuous anesthetic monitoring included heart and respiratory rates, rectal temperature, pulse oximetry, and capnography. The dorsum was washed, shaved, and cleaned with 98% ethanol, and a commercial dispersive electrode (Covidien) was placed. After application of three cycles of a 10% povidone-iodine solution and ethanol to the ventral abdominal skin, the liver was exposed through a midline incision. A commercial TSL-Dox formulation (ThermoDox^®^, Celsion Corp.) was infused intravenously over 30 minute duration at a dose of 0.6 mg/kg. After the infusion was completed, a cooled needle RF electrode with 3 cm active length (Covidien Cool-Tip) was inserted into a liver lobe. RF ablation heating was performed with impedance-controlled power application algorithm (max 200 W), either for 12 min (n = 2) or 5 min (n = 2), in randomized fashion; there was >10 cm distance between electrode locations in the liver. 30 minutes after completion of the final ablation, the pig was euthanized by increasing isoflurane concentration to 5%, followed by exsanguination. The liver lobes were extracted and immediately frozen on dry ice, and then kept in a freezer at -80°C.

#### Fluorescence Imaging

The frozen liver lobes were cut centrally through the ablation zone, perpendicular to the electrode axis. Samples were thawed to room temperature, and fluorescence imaging at the doxorubicin emission peak was performed (520 nm excitation, 600 nm emission filter) by a fluorescence imaging system (Maestro 2, Perkin-Elmer). In addition, each sample was imaged photographically with white light illumination to depict the visible coagulation zone. A calibration curve was created based on liver samples spiked with known, varying amounts of doxorubicin (measured via LC/MS), based on the fluorescence of those reference samples. Based on a bi-exponential approximation of the calibration curve (see Supporting Information, S1 File), the fluorescence intensity of the liver samples was converted into drug concentration using the image processing software ImageJ v1.49, and visualized on a linear color scale. Based on the calibrated fluorescence images, drug concentration was determined along a path from the center outwards. This was done along different path directions every 0.64°, covering 360°. An average concentration profile was calculated from all paths using the boundary of the visible coagulation zone as reference location.

#### Statistical analysis

Individual data points consisted of concentration values located along a radial path with distance relative to the coagulation boundary. At each measured distance, data were averaged over all the radial paths to estimate the average concentration as a function of relative distance from the boundary. Two separate sets of results were available for the 5 minute ablation, and two sets for the 12 minute ablation. Visual inspection of the plotted data suggested the data could be adequately modeled using a piecewise linear regression model, assuming linearity between and outside of three breakpoints [25]. Separate segmented linear regression models were constructed for each heating duration to estimate concentration as a function of distance using the software R [26]. One model was constructed each for 5 min and 12 min data, both between the distances of −5 mm to +12.6 mm relative to the coagulation boundary. Break points were allowed vary the two models. Predicted results for “distance” were determined with their respective standard errors for each regression model, and estimated differences were calculated starting at − 5mm, and each +1 mm interval thereafter. Wald tests were used to determine if the differences were significantly different from zero at each point, using α = 0.05. To maintain a family-wise α = 0.05 using the Bonferroni method to adjust for multiple comparisons (n comparisons = 18), a p-value < 0.003 was considered significant. Detailed statistical models are presented in the Supporting Information (S1 File).

## Results

**Fig 2:**
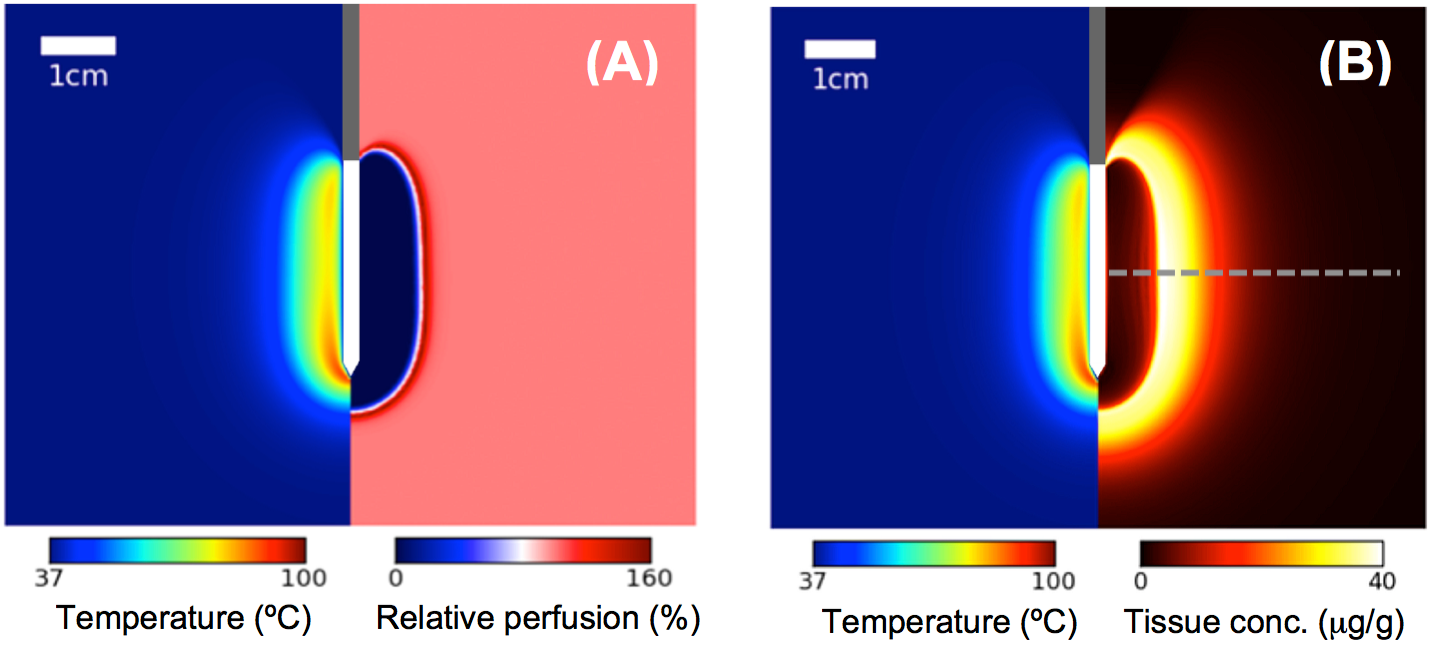
Computer model results. (A) Temperature (left half of figure) and perfusion map (right half of figure) at the end of a 12 min ablation. This perfusion map varied during heating depending on thermal dose (see supporting information, S1 File) (B) Temperature at the end of a 12 min ablation (left half of figure), and total tissue doxorubicin concentration 30 min after completion of ablation (right half of figure). The RF heating electrode is located at the center (gray: insulated catheter region; white: active tip region). The dashed line indicates the location where concentration profile is plotted in Fig 4, and also indicates approximate slice location of *in vivo* tissue slices in Fig 3.

All tissue drug concentrations based on computer models were calculated 30 min after discontinuation of heating to allow for tissue cooling and any additional drug uptake. After 12 min ablation, the central ablation zone is devoid of perfusion, with a hyper-perfused rim surrounding the ablation zone (Fig 2A). The cessation of perfusion in the central coagulated region inhibits TSL delivery to this zone, resulting in limited drug deposition (Fig 2B). The highest doxorubicin concentration is found in the margin of the ablation zone where temperatures in the range of 40–50°C are obtained for extended duration (Fig 2B).

**Fig 3:**
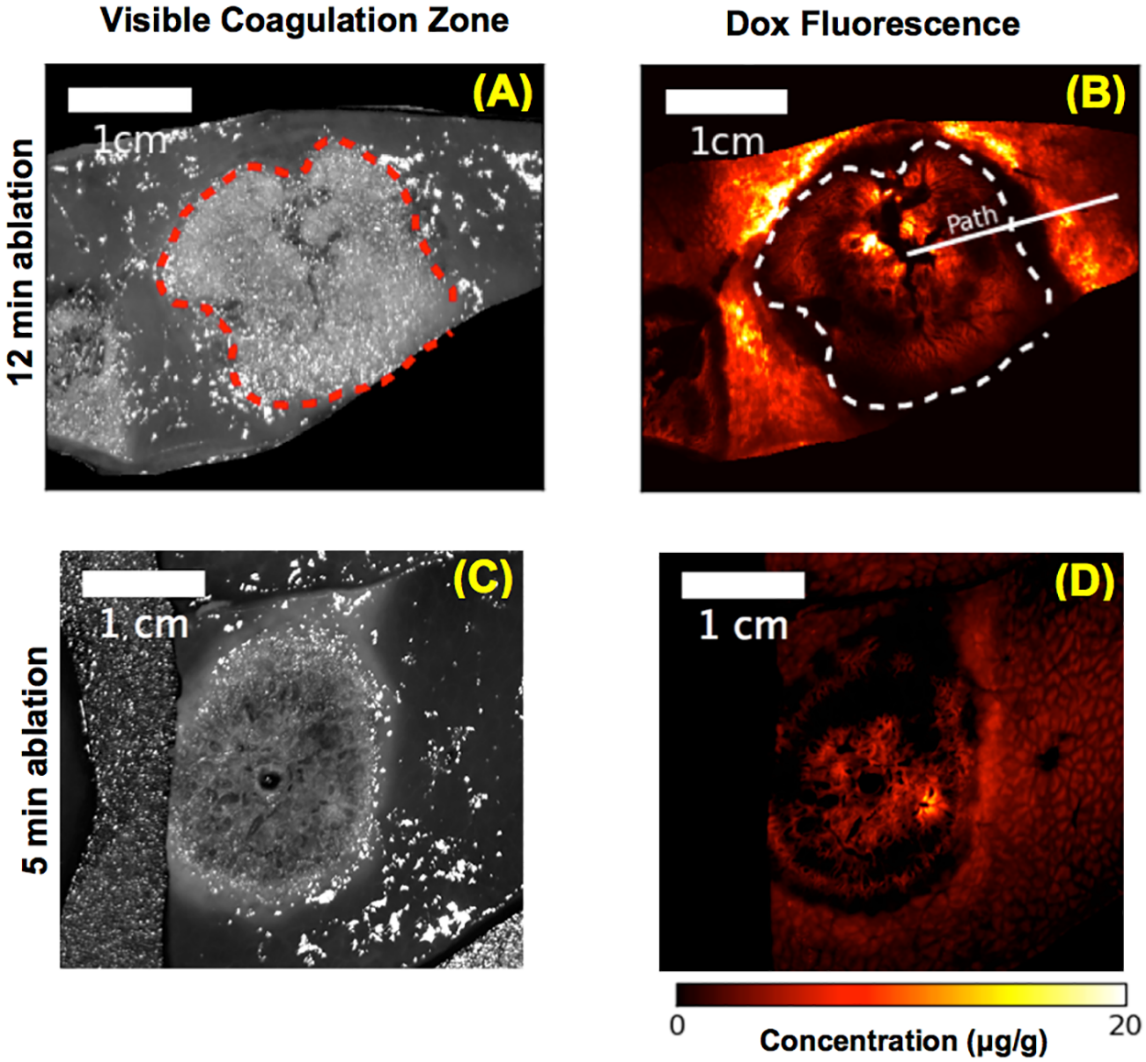
*In vivo* results. Grey scale images demonstrate the visible coagulation zone (red dashed line in (A)) after either 12 min (A), or 5 min (C) ablation. The color images show tissue doxorubicin concentration extracted from fluorescence imaging of the same tissue slices after either 12 min (B), or 5 min (D) ablation. The visible coagulation zone is marked by dashed lines in (A) and (B). A radial sample path along which drug concentration profile was calculated is shown in (B). Note these slices are oriented orthogonal to the computer simulation results (dashed line in Fig 2B).

Similar to computer models, *in vivo* results demonstrate doxorubicin delivery within the margin of the ablation zone (Fig 3).

**Fig 4:**
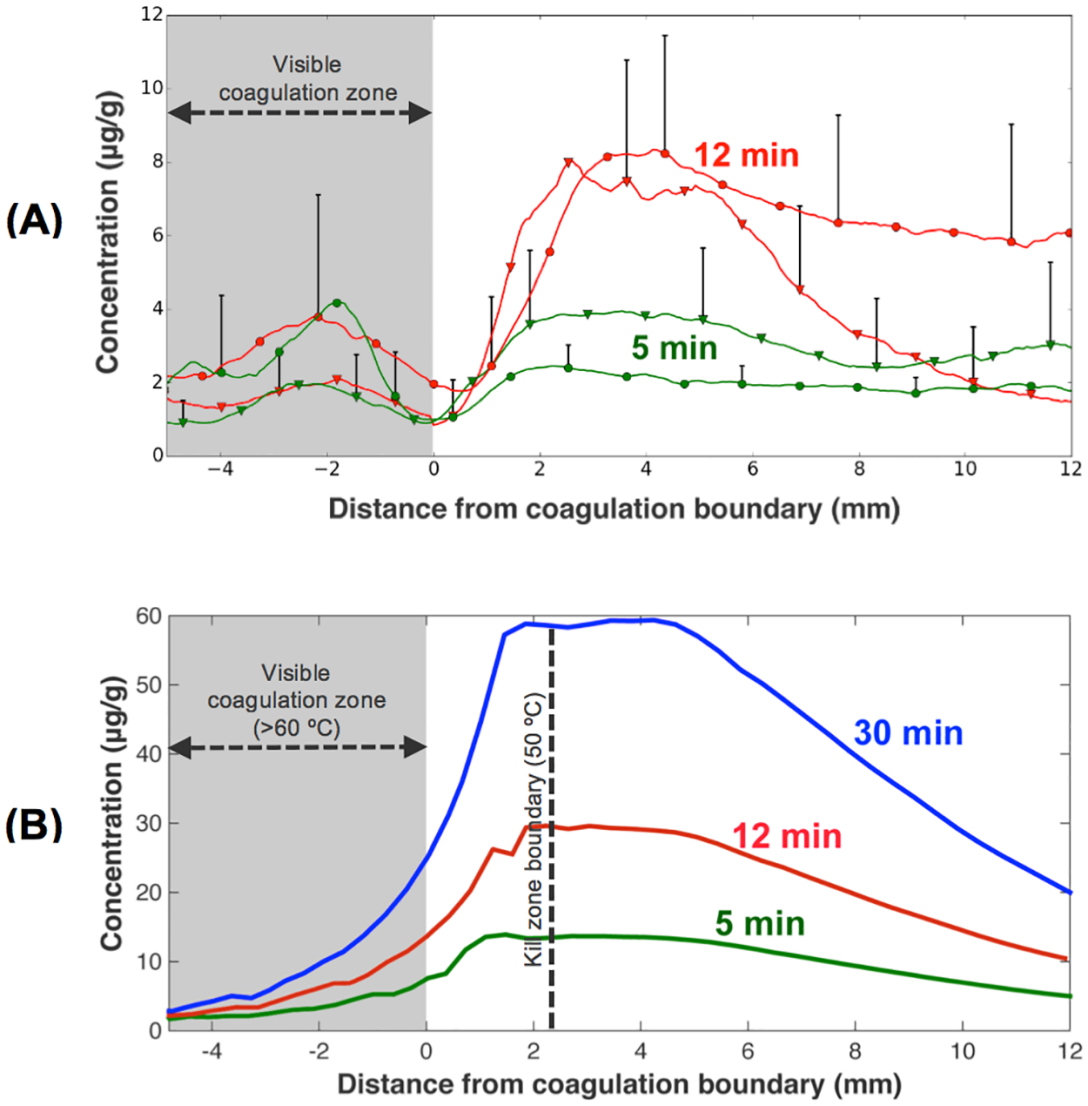
Radial drug concentration profile. (A) *In* vivo radial drug concentration profile (see path in Fig 5B, averaged over 360°), referenced to the boundary of the visible coagulation zone. Two heated tissue samples each were evaluated for 5 and 12 min ablation, indicated by circles and triangles. There was statistically significant difference between the two groups (5, 12 min) from 2 to 12 mm distance. (B) Radial drug concentration from computer simulation (calculated 30 min after ablation completion). Drug uptake increases approximately linearly with ablation time for both *in vivo* study and computer model. Most of the drug is delivered to within ~10–15 mm of the ablation zone boundary. Note that the visible coagulation zone indicated by the gray shaded region in (A) and (B) does not represent the tissue region destroyed by heat. The boundary of the kill zone (= ablation zone = region destroyed by heat) is indicated by a dashed line in (B), and extends ~2.2 mm beyond the visible coagulation zone. I.e. there is no viable tissue zone in-between the ablation zone and the region of drug delivery, as may be assumed from Figs 5 and 6A.

The amount of doxorubicin delivered to the ablation margin increases with hyperthermia duration, both *in vivo* and in computer models (Fig 4).

We compared average drug concentration in the region of maximum concentration (~2–4 mm from coagulation boundary): in the computer simulations, drug concentration increased by a factor of 2.2x and 4.3x when heating duration was extended from 5 min to either 12, or 30 minutes, respectively. *In vivo*, drug concentration was by a factor of 2.4x higher at 12 min vs. 5 min heating duration (7.1 μg/g vs. 3.0 μg/g (average between 2–4 mm)). Note that the visible coagulation zone (Fig 4A and 4C) underestimates the actual ablation zone (i.e. tissue destroyed by heat). In Fig 4B the visible coagulation zone and ablation zone boundary (zone of tissue kill) are both indicated. We assumed that visible coagulation occurs at 60°C [27], while tissue is ablated (i.e. killed by heat) above 50°C [28]. While it is known that cell death depends both on temperature and time (i.e. thermal dose), prior modeling studies suggest that the 50°C boundary is an adequate predictor of the kill zone for ablation therapies [29, 30].

**Fig 5:**
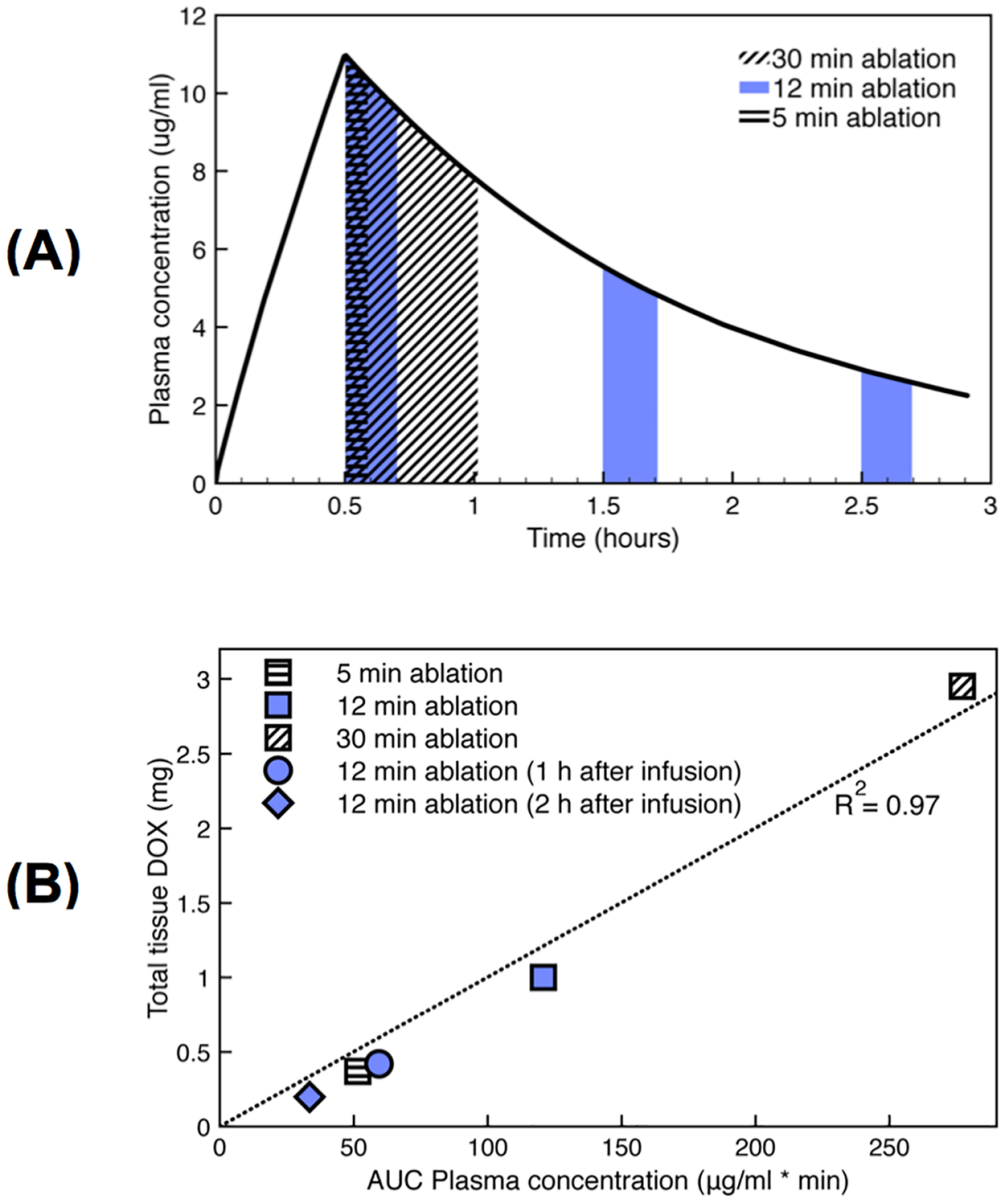
Plasma AUC predicts tissue uptake. Computer simulation results: (A) Systemic plasma concentration of TSL-Dox (i.e. encapsulated Dox). Areas under the curve (AUC’s) are colored/shaded for an ablation heating cycle initiated immediately following a 30 min TSL-Dox infusion (5, 12, and 30 min duration), or following 1 and 2 hours after infusion (12 min duration). (B) Systemic plasma AUC of TSL-Dox correlates with total amount of doxorubicin delivered to the target tissue (R^2^ = 0.97).

The amount of drug delivered further depends on, when ablation is performed relative to the time of TSL-Dox administration, with RF heating ideally starting immediately after administration (Fig 5).

The area under the curve (AUC) of the systemic concentration of encapsulated Dox, calculated during the time RF heating was applied, showed excellent correlation with total amount of drug delivered to the tissue (Fig 5). The total amount of drug was calculated by summing drug concentration throughout tissue regions where drug concentration exceeded 1 μg/g, i.e. where our data suggests that significant drug uptake occurred (for comparison, in unheated tissue regions, drug concentration was in the range of 0.1–0.2 μg/g).

**Fig 6:**
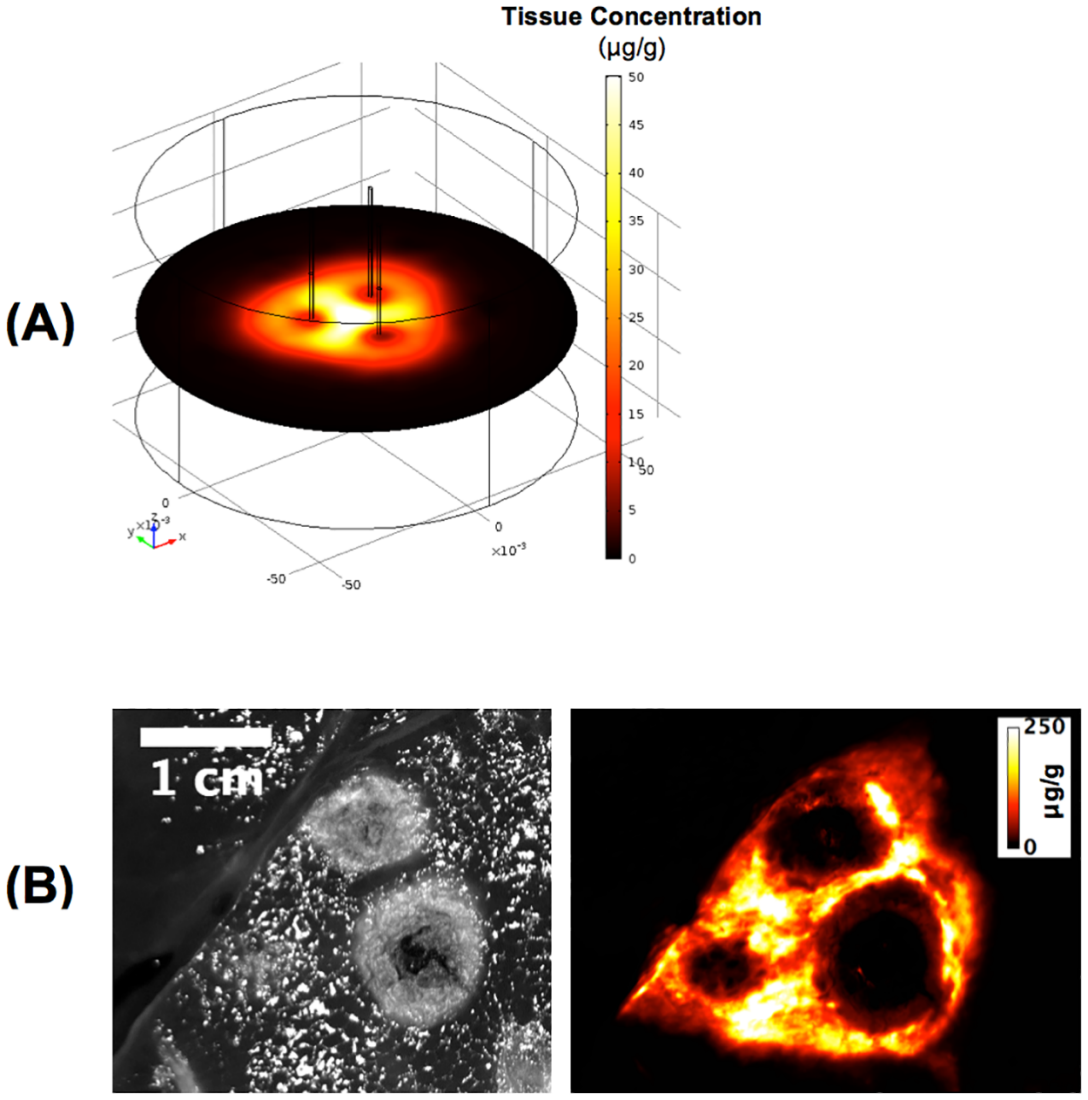
Heating with multiple RF electrodes. (A) 3-D computer model results for three-electrode array, where a 12-min ablation was performed with each needle sequentially to emulate clinical treatment of a large tumor by multiple overlapping ablations. Drug delivery is enhanced in tissue regions that were exposed to hyperthermic temperatures by more than one heating cycle, i.e. the areas in-between the RF needles. This observation is clinically relevant, as this preferential delivery may prevent tumor recurrence in untreated tumor regions that remain when sequential ablations are not overlapping. (B) A prior *in vivo* study demonstrates a similar drug delivery pattern with enhanced uptake in-between electrodes after three sequential ablations (3 x 12 min) [31]. Left image shows the visible coagulation zone, right image shows drug fluorescence. This prior study used smaller electrodes (resulting in smaller ablations), and used a ~2.3 times higher TSL-Dox dose (1.43 mg/kg), than the current study, explaining the substantially higher tissue drug concentration. (images in (B) produced base on prior data [31]).

When multiple tissue regions were heated sequentially via three separately placed RF electrodes (Fig 6), enhanced drug delivery was observed between the electrodes, as these regions were exposed to hyperthermic temperatures two or three times (i.e. during 2–3 of the three heating cycles).

## Discussion

Temperature sensitive liposomes (TSL) are triggered drug delivery systems, that release the contained agent when exposed to temperatures above typically ~40°C [17, 32]. Recent studies demonstrate that the newer TSL formulations that release very rapidly (within seconds) are based on an intravascular triggered release mechanism [5–7], where drug is released and then extracted by tissue as TSL pass through the vasculature of the heated region. The current study employs one of these rapid-release TSL formulations filled with doxorubicin (TSL-Dox) that is currently in clinical trial [14].

Here, we combine TSL-Dox with radiofrequency (RF) ablation—a clinically employed localized cancer therapy that is widely used for liver cancer [8]. RF ablation creates a central zone of cytotoxic temperatures (>50°C), surrounded by a mildly hyperthermic zone (<50°C) (Fig 2). The combination of RF ablation with TSL delivers drug to the margin of the ablation zone (i.e. the zone directly killed by heat) (Figs 2 and 3). This combination is of clinical relevance, since local tumor recurrences after RF ablation typically occur at this margin [11, 33–36]. There are numerous other studies where TSL have been combined with other heating modalities such as focused ultrasound, microwave, or laser to achieve localized drug delivery [20] [37] [38].

There is no general agreement on the timing at which heating should ideally be performed relative to administration of TSL-Dox. Some published studies pre-heat tissue before administering TSL-Dox [7, 18, 20, 38, 39], others initiate heating following drug administration [2, 6, 15, 37]. In a clinical Phase I trial where RF ablation was combined with TSL-Dox, RF heating was initiated midway through a 30-min drug infusion [14]. Similarly, the ideal heating duration is not known. Some small animal studies, where RF heating or focused ultrasound heating were combined with TSL, employed short heating durations of 2–3 min [15, 16]. Several other studies used much longer heating durations, between 30 and 60 min [2, 6, 7, 17–20]. There is a lack of studies where the effects of heating duration and/or timing relative to drug administration are investigated. In one study where multiple adjacent tissue volumes were heated by sequential RF ablation applications, an increase in drug delivery was demonstrated, but it is not clear whether this enhanced delivery was from enlarging the heated tissue volume, or from increased duration of heating [31].

Here, we used computational models simulating RF heating combined with a multi-compartmental drug delivery model (Fig 1) [1], to identify the effects of varying heating duration and timing on amount of drug delivered to the target site. The model simulated tissue heating due to RF energy, and included temperature dependent changes in perfusion. Temperature and perfusion served as input data to the drug delivery model, which considered temperature-dependent drug release from TSL-Dox, trans-vascular transport of released drug, and cellular drug uptake (Fig 1) [1, 5, 40].

The computer model results demonstrate an almost linear increase in drug delivery with heating duration between 5 and 30 minutes (Fig 4B). The model results are supported by *in vivo* studies in normal porcine liver, where RF ablation was applied either for 5 or 12 minutes (Fig 4A). The reason for this direct dependence of drug delivery on heating duration is that the rapid-release TSL used here are based on intravascular triggered release mechanism. That means, as TSL enter the vasculature of the heated region, drug is released within the vasculature and immediately taken up by tissue [5, 7]. This process continues as long as hyperthermia is applied, and thus longer heating results increased drug release and—uptake.

Both computer simulation and *in vivo* studies demonstrate a ring-shaped delivery region of drug just outside the visible coagulation zone (Fig 3). While it appears that there may be a viable tissue region between the visible coagulation zone and the drug delivery region (Figs 3 and 4A), visible tissue coagulation occurs at higher temperatures and thermal dose than required for ablation (i.e. thermal tissue destruction) [27, 28, 41], and the computer model suggests that no viable tissue regions remain (see dashed line, Fig 4B).

The amount of Dox locally released from TSL within the vasculature is determined by available encapsulated TSL-Dox within the plasma. During heating, drug-loaded TSL continuously enter the vasculature of the hyperthermic region and release drug; i.e. the systemic plasma volume serves as reservoir of non-bioavailable drug that becomes bioavailable once entering the heated region. Thus, the AUC of encapsulated TSL-Dox concentration within the systemic plasma, calculated during the heating duration, corresponds to the total amount of Dox released in the heated tissue region (Fig 5A). Based on our computer simulations, this AUC showed excellent correlation with total amount of drug delivered to the target region for different heating durations and timings (Fig 5B). Thus, this AUC may serve as a simple parameter that can aid in optimizing heating and administration regimen for TSL based drug delivery. Notably, heating cycles initiated late after TSL-Dox administration resulted in considerably reduced drug delivery due to declining encapsulated TSL-Dox available in systemic plasma circulation. In the central tissue regions in proximity of the RF electrode, vessels are coagulated soon after initiation of heating. Without blood flow, no new TSL can enter these tissue regions to release drug. Thus very little Dox is deposited in the central, coagulated tissue regions (Figs 2 and 3).

In a three-dimensional model we simulated sequential ablation with three adjacent RF electrodes and demonstrate the resulting drug distribution (Fig 6). Notably, in the overlapping regions in-between electrodes that are exposed to hyperthermic temperatures during two or three heating cycles, drug uptake is accordingly enhanced. This observation is clinically relevant, as this preferential delivery may prevent tumor recurrence in untreated tumor regions that remain when sequential ablations are not sufficiently overlapping.

Our results may in part explain the limited efficacy of TSL-Dox in some studies where short heating duration (<5 min) was employed [15]. Further, this study demonstrates that the dose locally delivered to the target site can be controlled by heating duration. This ability to control both the location and the amount of drug delivered is unlike most other drug delivery systems, and may lead to novel treatment paradigms.

Finally, this study demonstrates the ability of computer models to predict spatial drug delivery dynamics, and such models may serve as a powerful tool in understanding and optimizing drug delivery systems.

### Study limitations

This study was performed in normal liver. Perfusion, vascular permeability, cell uptake rates and other parameters are likely different in tumors, and may affect drug delivery. Further, the size of porcine liver was in some cases insufficient to completely encompass the ablation zone (Fig 3), possibly affecting drug uptake. The computer model predicts ~2 to 3 times higher tissue drug concentration than the experimental results. Possible contributing factors include incorrect model parameters (e.g. perfusion, plasma volume, TSL release properties, cell uptake), effects of inadequate liver size noted above, and potential errors resulting from conversion of fluorescence to tissue drug concentration.

## Conclusions

Tissue drug uptake directly correlates with hyperthermia duration. Computer models suggest that AUC of plasma concentration of TSL-Dox calculated during heating is predictive of drug delivery. The computational models were able to predict the spatial drug delivery profile, and may thus serve as a valuable tool in understanding and optimizing drug delivery systems.

## Supporting information

S1 File Contains complete information on computer models (equations and parameters), details on statistical analysis, and additional in vivo tissue fluorescence images. (PDF)
