# Supplementary material for: Temperature sensitive liposomes combined with thermal ablation: Effects of duration and timing of heating in mathematical models and in vivo
Source: PLoS One. 2017 Jun 12;12(6):e0179131. doi: 10.1371/journal.pone.0179131 (PMC5467840; doi:10.1371/journal.pone.0179131)
Supplement: S1 File — (PDF) [file pone.0179131.s001.pdf]

Supporting Information

Computer model

Mathematical model of radiofrequency heating

Similar to prior studies, we coupled a heat-transfer model simulating RF ablation heating with a drug delivery model, and simulated these in 3-D via Finite Element Method [1, 2]. In the heat-transfer model, the temporally and spatially varying tissue temperature profile resulting from RF heating was calculated by solving Pennes' Bioheat equation [3]. Perfusion is altered in response to hyperthermic and ablation range temperatures [4], and thus affects both RF tissue heating and drug delivery. In prior modeling studies [2], hyperthermia induced perfusion change was modeled based on small animal tumor studies [5]. Since here we modeled normal liver tissue, we considered perfusion temperature dependence based on measurements in normal porcine kidney [6], as similar data on liver was not available. Detailed equations and parameter values are listed in the Supplementary Materials.

The main governing equation that has established to model the temperature distribution in biological tissues is the Bioheat equation and is based on the general equation for conservation of energy [3]:

$$\rho c \frac{\partial T}{\partial t} = k\nabla^2 T + Q_{\mathrm{rf}} - Q_{\mathrm{P}} \,, \qquad (1)$$

ρ is the density ($\mathrm{kg \cdot m^{-3}}$), c is the specific heat ($\mathrm{J \cdot kg^{-1} \cdot K^{-1}}$), k is the thermal conductivity ($\mathrm{W \cdot m^{-1} \cdot K^{-1}}$), Q_{rf} ($\mathrm{W \cdot m^{-3}}$) is the heat generated by RF current, and Q_{P} ($\mathrm{W \cdot m^{-3}}$), is the heat loss due to blood perfusion. The energy generated by the metabolic processes is neglected since it is orders of magnitude lower than Q_{P}.

$$Q_{\mathrm{P}} = \rho_{\mathrm{bl}} c_{\mathrm{bl}} w_{\mathrm{bl}} (\mathrm{T} - \mathrm{T_{bl}}), \qquad (2)$$

T_{bl} is the temperature of the blood (commonly assumed to be 37 °C), ρ_{bl} is the blood density ($\mathrm{kg \cdot m^{-1}}$), c_{bl} is the specific heat of human blood ($\mathrm{J \cdot kg^{-1} \cdot K^{-1}}$), and w_{bl} is the blood perfusion ($\mathrm{s^{-1}}$). The temperature dependence of the blood perfusion term w_{bl} was implemented as described in a prior publication [7], with an initial increase in perfusion followed by vascular stasis (Fig. S1). This temperature dependence was implemented based on a first-order kinetic Arrhenius model. A variable "degree of stasis" (DS) was modeled according to following equation with A as the frequency factor (1.98×10^{106} $\mathrm{s^{-1}}$), and ΔE is the activation energy (6.67×10^{5} $\mathrm{J \cdot mole^{-1}}$) [7]:

$$DS = 1 - \exp - \int_0^t A e^{-\Delta E / [RT(\tau)]} d\tau \qquad (3)$$

Based on DS, perfusion was varied according to Figure S1.

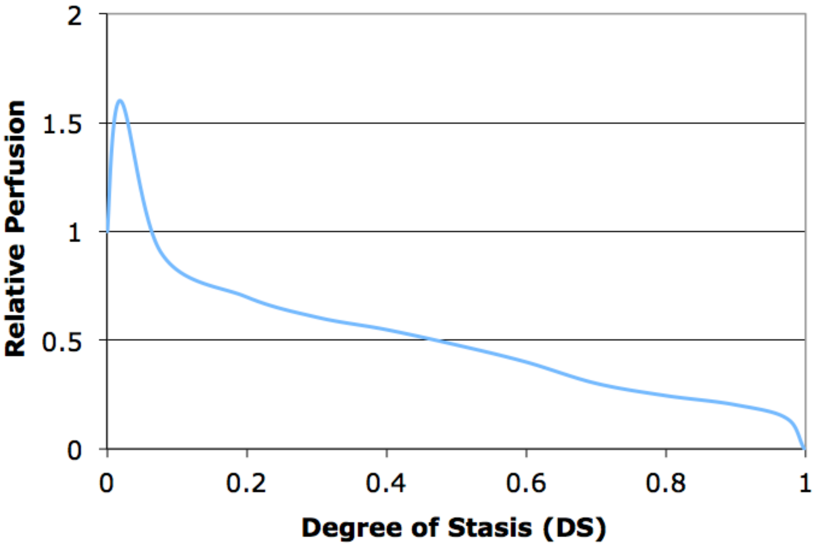

Fig. S1. Relative perfusion vs. degree of stasis (DS) used to model dose dependent perfusion change.

For RF heating at frequencies between 300 kHz and 1 MHz, tissue can be considered as purely resistive as the as the displacement currents are negligible. Thus heating around the active electrode due to dissipating electrical power Q_h (Joule loss) can be modeled via quasi-static approach:

$$Q_{rf} = J \cdot E, \qquad (4)$$

where J (Am^{-1}) is the current density and E (Vm^{-1}) is the electric field intensity [8, 9] which can be evaluated from the Laplace's equation $\nabla \cdot (\sigma \nabla V) = 0$, thus Q_h can be expressed as:

$$Q_{rf} = \sigma |\nabla V|^2, \qquad (5)$$

where σ is the electrical conductivity and V is the electric potential (Volts).

Table S1. Parameters of heat-transfer model.

Symbol	Description	Value	Source
ρ	Mass density of tissue	1060 kg m^{-3}	[10]
c^*	Specific heat of tissue	3600 J kg^{-1} K^{-1}	[10]
k^*	Thermal conductivity of tissue	0.52 W m^{-1} K^{-1}	[10]
σ^*	Electrical conductivity	0.333 S m^{-1}	
c_{bl}	Specific heat of blood	3800 J kg^{-1} K^{-1}	[10]
w_{bl}	Blood perfusion rate	0.018 s-1	[11]
T_{bl}	Arterial blood temperature	37 ºC	NA
k_p	Controller parameter (proportional term)	0.2	NA
k_i	Controller parameter (integral term)	0.01	NA

In Table 1, temperature dependence of electrical conductivity of liver tissue was assumed with 1.6 ºC^{-1} [12]. Temperature dependence of the thermal conductivity of liver tissue was implemented according to *ex-vivo* measurements from a prior study [13]. The value of latent heat of vaporization for water was used for liver tissue as in previous studies [14, 15].

Mathematical drug delivery model

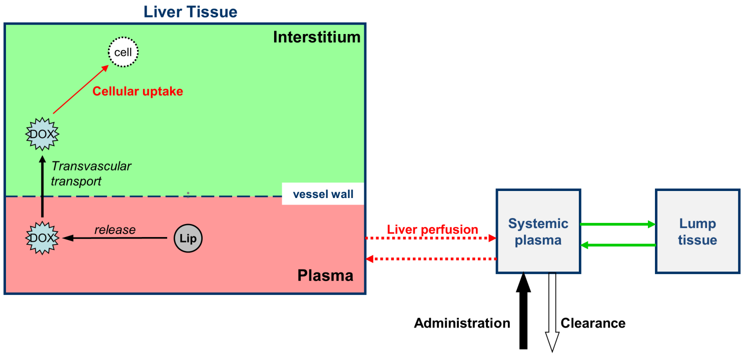

Fig. S2. Drug delivery model overview. Intravascular temperature-dependent release of Dox from TSL, uptake by interstitium (EES), and cell uptake are simulated. Local temperature and perfusion are fed into the drug delivery model from the heat transfer model.

The tissue temperature and perfusion were fed into the drug delivery model to calculate intravascular release from TSL-Dox, transport from plasma into tissue interstitium (extracellular-extravascular space (EES)), and uptake by liver cells (Fig. S2), similar to prior studies [1, 2]. Similar to prior models, the targeted liver tissue and body compartments were modeled separately [2]. Liver tissue was modeled via three compartments: plasma, extracellular-extravascular space (EES), and intracellular space. The body was modeled via tissue compartment (representing all body tissues except tumor i.e. organs with a significant drug uptake) and plasma compartment. These systemic plasma and tissue compartments were not considered spatially varying. In order to simulate spatio-temporal release and transport of Dox the tumor was modeled by spatially varying compartments; i.e. each location within the tumor was represented by its own sub-compartments. The normal tissue was represented by a single compartment without considering spatial variation and the systemic plasma compartment included the total blood plasma volume with exception of the tumor. The administered TSL-Dox at a dose of 30 mg is modeled as a 30 min continuous infusion into the systemic plasma compartment. Pharmacokinetics (PK) of Dox after release was based on prior studies [16], as were PK and release kinetics of TSL-Dox [2, 17]. Note that we considered uptake kinetics of normal liver cells [18] rather than tumor cells as in prior studies [2], to allow direct comparison to the *in vivo* studies that were performed in normal porcine liver.

We used equations from Gasselhuber *et al.* [2] to model the transport of Dox from the systemic plasma compartment with the normal tissue compartment and with the tumor plasma space, and from the tumor plasma transport of Dox into the tumor interstitium. Gasselhuber *et al.* used a cell uptake model developed El-Kareh *et al.* [19], based on in vitro studies on lung cancer cells [20]. Since in the current study the computer model

was compared to *in vivo* studies in normal liver, a cell uptake model for normal liver cells was used, based on prior *in vivo* studies [18]. In addition, we added a source term in equation (5) to model infusion, rather than bolus administration as in the prior model [2].

$$\frac{dc_{p\_TSL}}{dt} = \frac{D/V_p^B}{T_{\inf}} - k_{e\_TSL}c_{p\_TSL} - R_{R37}c_{p\_TSL} \qquad (6)$$

TSL-Dox concentration in systemic plasma (encapsulated drug)

$$\frac{dc_p^B}{dt} = \frac{\int_V (F_{pv}^T c_p^T v_p^T)\,dV}{V_p^B} + c_{p\_TSL}R_{R37} - k_e c_p^B - k_p c_p^B + k_t c_t^B - c_p^B \frac{\int F_{pv}^T\,dV}{V_p^B} v_p^T \qquad (7)$$

Dox concentration in systemic plasma (unencapsulated drug)

$$\frac{dc_t^B}{dt} = k_p c_p^B - k_t c_t^B \qquad (8)$$

Dox concentration in systemic tissue

$$\frac{dc_p^T}{dt} = -\frac{1}{v_p^T} PS\cdot(c_p^T - c_e^T) - F_{pv}^T c_p^T + F_{pv}^T c_p^B + c_{p\_TSL}R_R \qquad (9)$$

Dox concentration in liver plasma

$$\frac{dc_e^T}{dt} = \nabla\cdot(\mathrm{Diff}\cdot\nabla c_e^T) + \frac{1}{v_e^T} PS\cdot(c_p^T - c_e^T) - k_1 c_e^T + k_2 c_{i.u}^T \qquad (10)$$

Dox concentration in liver EES

$$\frac{dc_{i,u}^T}{dt} = k_1 c_e^T - k_2 c_{i,u}^T - k_3 c_{i,u}^T \qquad (11)$$

Dox concentration in liver cells (unbound)

$$\frac{dc_{i,b}^T}{dt} = k_3 c_{i,u}^T \qquad (12)$$

Dox concentration in liver cells (bound)

Within the EES, spatial diffusion was considered based on diffusion coefficients experimentally measured in liver tissue [21].

Table S2. Parameters for drug delivery model.

Symbol	Description	Value	Source
BW	Body weight	46.7 kg	to match *in vivo*
D	Administered dose	30 mg	to match *in vivo*
T_{inf}	Duration of infusion	30 min	to match *in vivo*
Hct	Hematocrit	0.45	[22]
Hct_{tumor}	Hematocrit for liver microvasculature	0.19	[23]
k_p	Transfer constant from systemic plasma to tissue	9.4e-3 s^{-1}	[24]
k_e	Transfer constant for clearance	2.1e-3 s^{-1}	[24]
k_t	Transfer constant from tissue to systemic plasma	7.052e-5 s^{-1}	[24]
k_{e_TSL}	Rate constant of TSL clearance	2.228e-4 s^{-1}	fit to data from [25]
k_1	Rate constant for intracellular uptake	0.24 s^{-1}	[26]
k_2	Rate constant for intracellular outflow	0.4 s^{-1}	[26]
k_3	Rate constant for intracellular binding	0.08 s^{-1}	[26]
PS	Permeability surface area product for Dox	4.9e-3 s^{-1}	[24]
V_B^B	Total blood volume in body	3.57 L	calculated with: blood = 7.5% of body weight [26]
V_p^B	Volume of systemic plasma	1.96 L	V_B^B(1-Hct)
V_{tissue}	Volume for body tissue (organs, etc.)	44.03 L	calculated with: blood = 7.5% of body weight [26]
v_v^l	Volume fraction of liver vascular space	0.092	[27]
v_p^l	Volume fraction of liver plasma space	0.07452	$v_v^l(1-Hct_{tumor})$
v_e^l	Volume fraction of liver EES	0.454	[28]
v_i^l	Volume fraction of liver intracellular space	0.454	$(1-v_v^l-v_e^l)$
R_R	Release rate of Dox from TSL	variable [s^{-1}]	[2]
R_{R37}	Release rate of Dox from TSL at 37 °C	variable [s^{-1}]	[2]
rf	Release fraction of Dox from TSL	variable	biexponential fit
F_{pv}^l	Plasma Flow in liver plasma<br>note: F_{pv}=plasma flow/plasma volume	variable [s^{-1}]	Calculated with heat transfer model
T	Temperature	variable [°C]	Calculated with heat transfer model
$Diff$	Diffusion coefficient for ablated tissue<br>Diffusion coefficient for non ablated tissue	1.1e-7 cm^2/s<br>6.7e-7 cm^2/s	[21]

Release rate calculation

The *in* v*itro* measured TSL release data in Fig. S3 was modeled by a bi-exponential fit (represented by solid lines). The drug fraction released from TSL depends on both local temperature, and on the time the plasma requires to pass through the heated tissue volume (=transit time, t_T) – i.e. the time TSL are exposed to heat. This transit time (t_T) depends on plasma perfusion as follows:

$$t_T = \frac{1}{F_{pv}^T}$$

The release rate is then calculated as follows, where rf(T,t_T) is the release fraction, i.e. fraction of drug released at a particular temperature (T), after time (t_T), corresponding to the data plotted in Fig. S3:

$$R_R(T, F_{pv}^T) = \frac{rf(T, t_T)}{t_T} = rf(T) \cdot F_{pv}^T$$

For a specific temperature, the release rate was interpolated between the temperatures where data was measured (see Fig. S3), by using linear interpolation of rf(T,t_T) between neighboring temperatures. The data for Fig. S3 was acquired in a prior study during *in vitro* studies, where TSL were immersed in plasma samples of varying temperatures, while measuring fluorescence of released drug. The method is described in more detail in the study where these data were initially published [2].

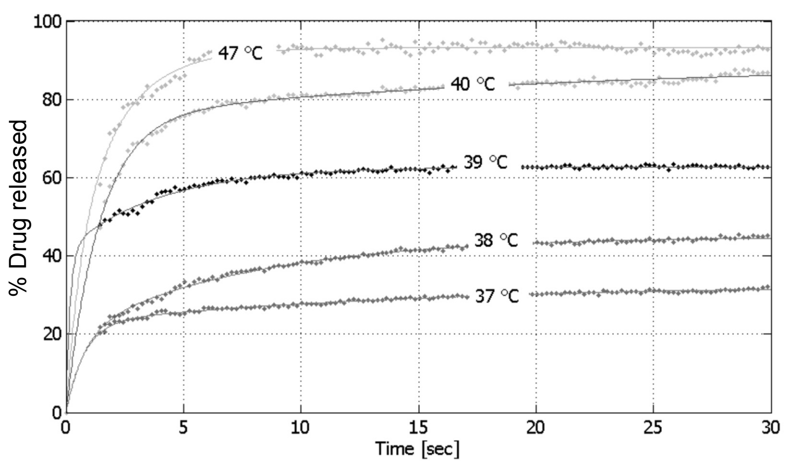

Fig. S3. TSL release kinetics. Drug release fraction, dependent on temperature and time, from *in vitro* measured data. Figure reproduced from Gasselhuber et al. [2]

Release at body temperature (37 ºC) was treated differently than described above. This was necessary since TSL are exposed to 37 ºC continuously after administration rather than just for a few seconds (i.e. transit time, t_T) as is the case for hyperthermic temperatures. Based on the derivative of the release curve for 37 ºC from Fig. S3, a temporally varying release rate $R_{R37}(t)$ was devised for equations (5) and (6).

Model geometry

Two model geometries were developed to simulate a single cooled needle electrode (model 1), and three cooled needle electrodes arranged in a triangular cluster, 2 cm apart (model 2, Fig. S4). Model 1 was employed to simulate RF ablation for 5, 12 and 30 minutes. In addition, a 12-minute ablation (the clinically used duration for this type of electrode) was simulated starting either immediately, 60 min, or 120 min after administration of TSL-Dox. In Model 2, three sequential 12-minute ablations were simulated, emulating clinical practice where multiple sequential ablations are employed to cover a large tumor.

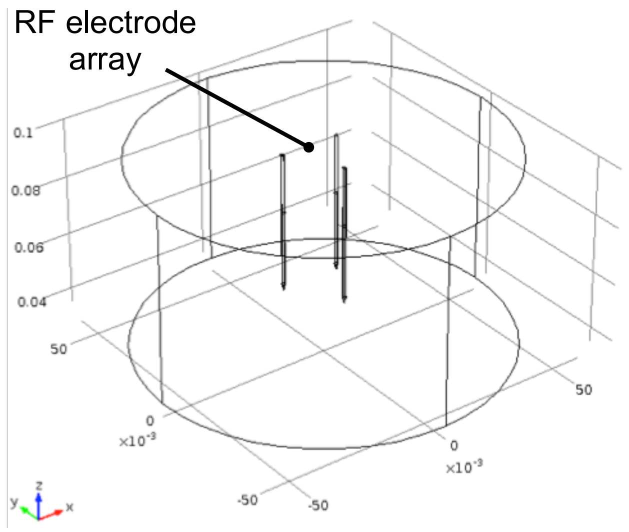

Figure S4. 3-D Model geometry. Three-electrode array (2 cm apart) is inserted into the tissue domain (diameter=12cm, height=6.5cm)

The commercial software Comsol 4.3 was used to simulate RF heating and drug delivery. For Model 1, a 2D-axially symmetric geometry was used, whereas Model 2 required a 3-D geometry (see Supplementary Materials). Model 1 consisted of ~20,000 triangular elements and model 2 consisted of 185,000 tetrahedral elements. Convergence tests were performed to ensure adequate mesh size. The temporal resolution for the models was 0.3–2 s.

Initial and boundary conditions

An initial temperature of 37 ºC was assumed throughout the model domain. Cooling of the needle electrode was simulated by setting electrode temperature constant to 20 ºC. Electric ground potential was assigned to the boundaries of the model domain, and V_{cc} was assigned to the active electrode tip. The voltage V_{cc} was varied throughout the simulation based on proportional-integral (PI) control algorithm to keep maximum tissue temperature at 100 ºC.

Calibration curve for conversion of fluorescence to doxorubicin concentration

Figure S5 shows the calibration curve used to convert fluorescence to tissue drug concentration. Since at very low concentrations the relationship between concentration and fluorescence is linear, we likely introduce an error at low concentrations (<~2 µg/g).

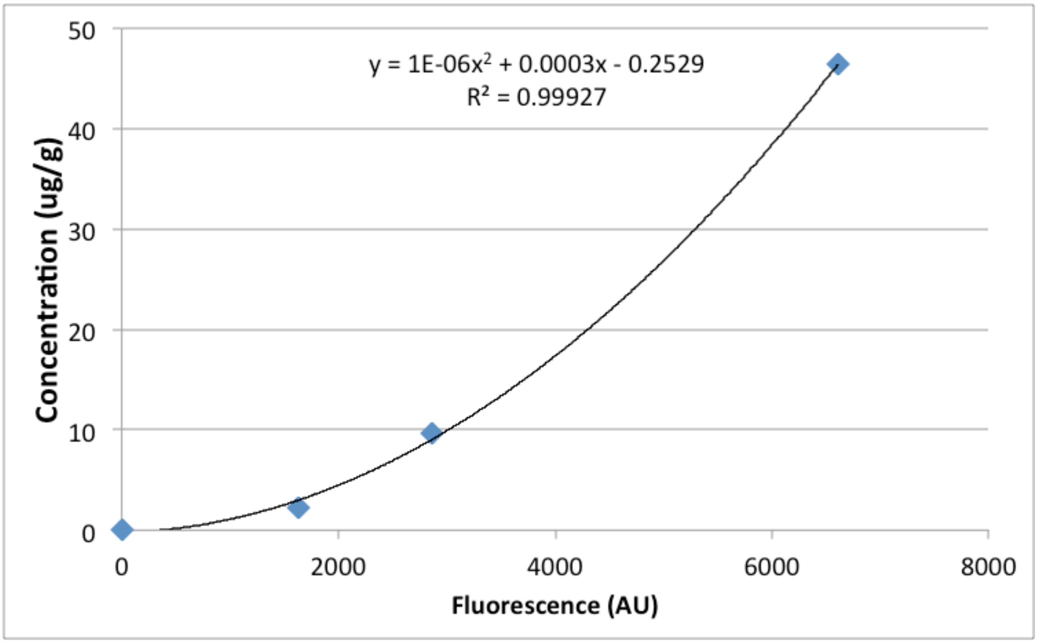

Fig. S5. Fluorescence calibration curve. A calibration curve was created based on four samples (blue diamonds) of known tissue doxorubicin concentration, and modeled by bi-exponential equation (black curve). This equation was employed to convert fluorescence intensity to tissue drug concentration.

Fluorescence images

Figure S6 shows fluorescence images of all 4 ablation samples.

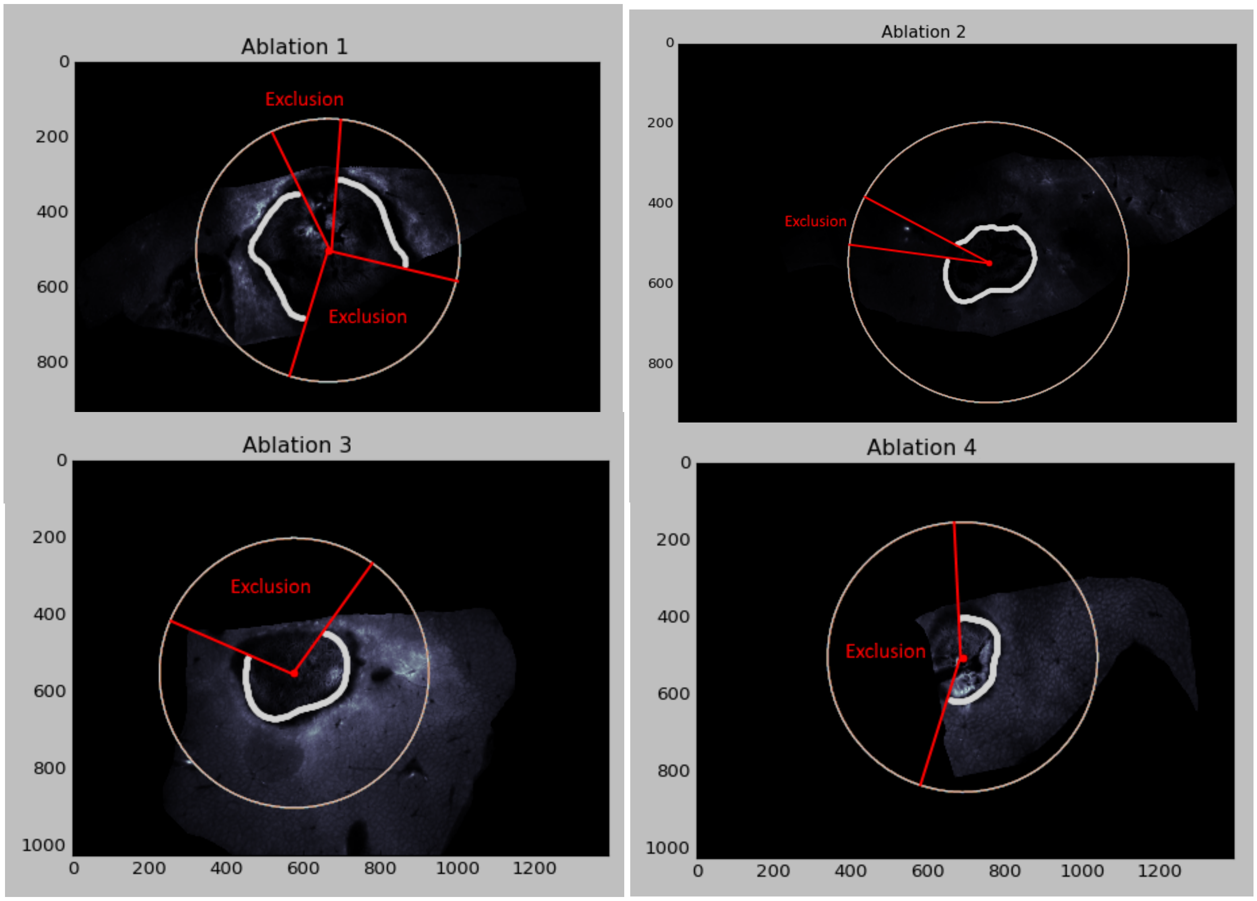

Fig. S6. Fluorescence images of all four ablations. Fluorescence is visualized in gray scale (12 min: ablation 1,3; 5 min: ablation 2,4). Angular segments were the visible coagulation zone was less than ~5 mm from the organ boundary were excluded from evaluation, indicated in each image by red angular sections. The voids in drug distribution are likely due to adjacent large vessels (see Figure S7).

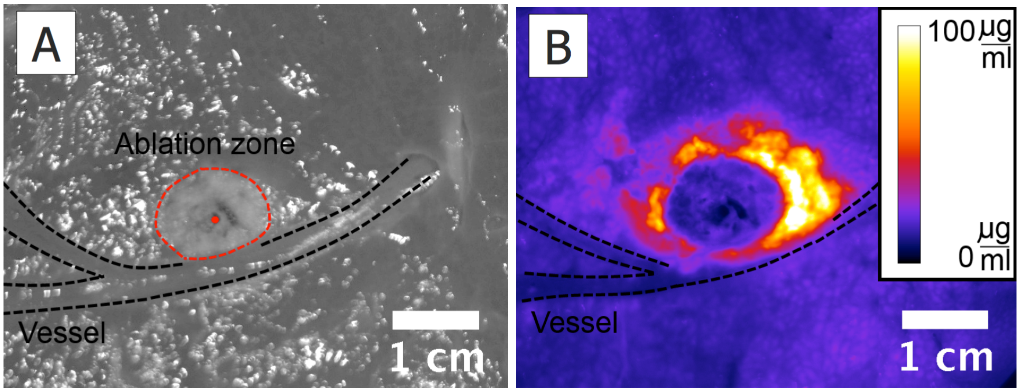

Fig. S7. Effect of proximal vessel on drug delivery. (A) Photographic image of an ablation close to a blood vessel. (B) Fluorescence image shows who the vessel reduces perivascular drug delivery, presumably due to vascular mediated cooling. Figure reproduced from Swenson et al. [29].

Statistical Analysis

Individual data points consisted of concentration values located along a radial path with distance relative to the boundary. At each measured distance, data were averaged over all the radial paths to estimate the average concentration as a function of relative distance from the boundary. Two separate sets of results were available for the 5 minute ablation, and two sets for the 12 minute ablation. Visual inspection of the plotted data suggested the data could be reasonably modeled using a piecewise linear regression model [30], assuming linearity between and outside of three breakpoints. Separate segmented linear regression models were constructed for each ablation time to estimate concentration as a function of distance using R [31], one model constructed for 5-minute data and one model for 12 minute data, both between the distances of -5mm to +12.6mm relative to the boundary (Fig. S8, Table 3). Break-points were allowed to be different between the two models. Predicted results for “Distance” were determined with their respective standard errors for each regression model, and estimated differences were calculated starting at -5mm, and each +1mm interval thereafter (Table 4). Wald tests were used to determine if the differences were significantly different from zero at each point, using $\alpha = 0.05$. To maintain a family-wise $\alpha = 0.05$ using the Bonferroni method to adjust for multiple comparisons (n comparisons = 18), a p-value < 0.003 would be considered significant.

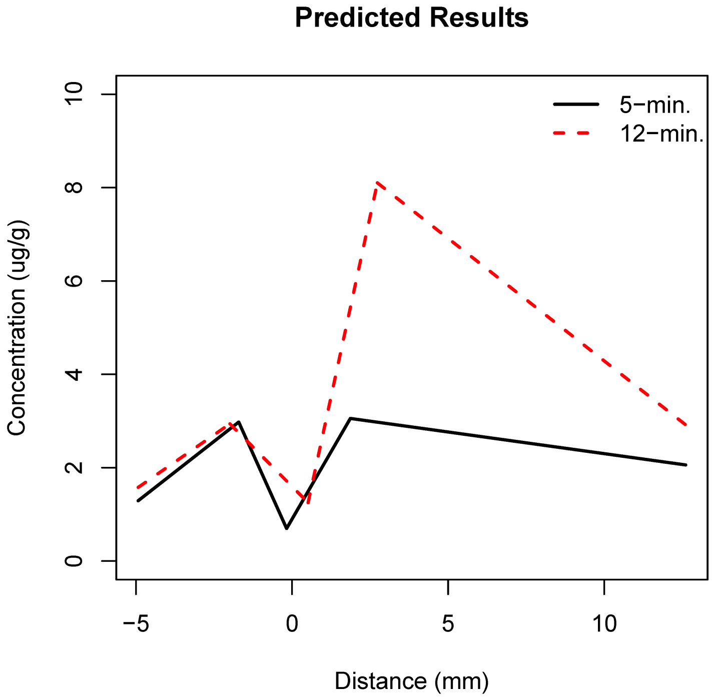

Fig. S8. Regression model predictions. Result curves predicted for 5 and 12 min based on the piecewise linear regression model.

Table S3. Regression Model Summary

	5-Minutes Estimate (Std. Error)	12-Minutes Estimate (Std. Error)
Model Regression Coefficients		
Intercept (β_0)	3.87 (0.249)	3.86 (0.570)
Distance (β_1)	0.52 (0.072)	0.46 (0.159)
U1.Distance (β_2)	-2.01 (0.236)	-1.14 (0.257)
U2.Distance (β_3)	2.64 (0.269)	3.76 (0.325)
U3.Distance (β_4)	-1.25 (0.147)	-3.61 (0.256)
Model Break-Points		
Point 1 (P_1)	-1.71 (0.119)	-1.99 (0.353)
Point 2 (P_2)	-0.17 (0.099)	0.52 (0.116)
Point 3 (P_3)	1.87 (0.152)	2.73 (0.098)
Model Segment Slopes		
Slope 1	0.52 (0.072)	0.46 (0.159)
Slope 2	-1.49 (0.225)	-0.68 (0.202)
Slope 3	1.15 (0.146)	3.09 (0.254)
Slope 4	-0.09 (0.012)	-0.53 (0.026)

To calculate estimated concentrations:
For distance $x_d < P_1$, Conc. = $\beta_0 + \beta_1 x_d$
For distance $P_1 < x_d < P_2$, Conc. = $\beta_0 + \beta_1 P_1 + (\beta_1 + \beta_2)(x_d - P_1)$
For distance $P_2 < x_d < P_3$, Conc. = $\beta_0 + \beta_1 P_1 + (\beta_1 + \beta_2)(P_2 - P_1) + (\beta_1 + \beta_2 + \beta_3)(x_d - P_2)$
For distance $x_d > P_3$, Conc. = $\beta_0 + \beta_1 P_1 + (\beta_1 + \beta_2)(P_2 - P_1) + (\beta_1 + \beta_2 + \beta_3)(P_3 - P_2) + (\beta_1 + \beta_2 + \beta_3 + \beta_4)(x_d - P_3)$

Table S4. Predicted values for 5 and 12 min ablation, and p-value for comparison at each 1 mm increment

Distance	Predicted 5-Minute	Predicted 12-Minute	Difference (12-5min)	P-value
-5	1.25	1.54	0.29	0.35
-4	1.78	2.01	0.23	0.20
-3	2.30	2.47	0.17	0.32
-2	2.82	2.93	0.11	0.71
-1	1.93	2.27	0.35	0.06
0	0.90	1.60	0.70	0.006
1	2.05	2.75	0.70	0.004
2	3.04	5.84	2.80	<0.001
3	2.95	7.96	5.01	<0.001
4	2.86	7.44	4.58	<0.001

5	2.76	6.91	4.15	<0.001
6	2.67	6.39	3.72	<0.001
7	2.58	5.86	3.28	<0.001
8	2.49	5.34	2.85	<0.001
9	2.39	4.81	2.42	<0.001
10	2.30	4.29	1.99	<0.001
11	2.21	3.76	1.55	<0.001
12	2.11	3.24	1.12	<0.001

References

1. Gasselhuber A, Dreher MR, Partanen A, Yarmolenko PS, Woods D, Wood BJ, et al. Targeted drug delivery by high intensity focused ultrasound mediated hyperthermia combined with temperature-sensitive liposomes: computational modelling and preliminary in vivo validation. Int J Hyperthermia. 2012;28(4):337-48. Epub 2012/05/25. doi: 10.3109/02656736.2012.677930. PubMed PMID: 22621735.
2. Gasselhuber A, Dreher MR, Negussie A, Wood BJ, Rattay F, Haemmerich D. Mathematical spatio-temporal model of drug delivery from low temperature sensitive liposomes during radiofrequency tumour ablation. Int J Hyperthermia. 2010;26(5):499-513. Epub 2010/04/10. doi: 10.3109/02656731003623590. PubMed PMID: 20377363; PubMed Central PMCID: PMC2958178.
3. Pennes HH. Analysis of tissue and arterial blood temperatures in the resting human forearm. Journal of applied physiology. 1948;1(2):93-122. Epub 1948/08/01. PubMed PMID: 18887578.
4. Rossmann C, Haemmerich D. Review of temperature dependence of thermal properties, dielectric properties, and perfusion of biological tissues at hyperthermic and ablation temperatures. Crit Rev Biomed Eng. 2015;in press.
5. Brown SL, Hunt JW, Hill RP. Differential thermal sensitivity of tumour and normal tissue microvascular response during hyperthermia. Int J Hyperthermia. 1992;8(4):501-14. PubMed PMID: 1402130; PubMed Central PMCID: PMCA.
6. He X, McGee S, Coad JE, Schmidlin F, Iaizzo PA, Swanlund DJ, et al. Investigation of the thermal and tissue injury behaviour in microwave thermal therapy using a porcine kidney model. International Journal of Hyperthermia. 2004;20(6):567-93. PubMed PMID: 15370815; PubMed Central PMCID: PMCAB.
7. Schutt DJ, Haemmerich D. Effects of variation in perfusion rates and of perfusion models in computational models of radio frequency tumor ablation. Med Phys. 2008;35(8):3462-70. Epub 2008/09/10. PubMed PMID: 18777906.
8. Haemmerich D, Webster JG. Automatic control of finite element models for temperature-controlled radiofrequency ablation. Biomedical engineering online. 2005;4(1):42. Epub 2005/07/16. doi: 10.1186/1475-925X-4-42. PubMed PMID: 16018811; PubMed Central PMCID: PMC1180460.
9. Tungjitkusolmun S, Staelin ST, Haemmerich D, Tsai JZ, Webster JG, Lee FT, Jr., et al. Three-Dimensional finite-element analyses for radio-frequency hepatic tumor ablation. IEEE transactions on bio-medical engineering. 2002;49(1):3-9. Epub 2002/01/19. doi: 10.1109/10.972834. PubMed PMID: 11797653.
10. Duck FA. Chapter 2: Thermal Properties of Tissue. Physical Properties of Tissue. London: Academic Press; 1990. p. 167-223.
11. Van Beers BE, Leconte I, Materne R, Smith AM, Jamart J, Horsmans Y. Hepatic perfusion parameters in chronic liver disease: dynamic CT measurements correlated with disease severity. AJR Am J Roentgenol. 2001;176(3):667-73. Epub 2001/02/27. PubMed PMID: 11222202.
12. Duck FA. Physical Properties of Tissue. Duck FA, editor. London: Academic Press; 1990. 336 p.

13. Valvano JW, Cochran JR, Diller KR. Thermal conductivity and diffusivity of biomaterials measured with self-heated thermistors. Int J Thermophys. 1985;6(3):301-11.
14. Haemmerich D, Chachati L, Wright AS, Mahvi DM, Lee FT, Jr., Webster JG. Hepatic radiofrequency ablation with internally cooled probes: effect of coolant temperature on lesion size. Ieee T Bio-Med Eng. 2003;50(4):493-500. Epub 2003/05/02. PubMed PMID: 12723061.
15. Yang D, Converse MC, Mahvi DM, Webster JG. Expanding the bioheat equation to include tissue internal water evaporation during heating. Ieee T Bio-Med Eng. 2007;54(8):1382-8. Epub 2007/08/19. PubMed PMID: 17694858.
16. Gustafson DL, Rastatter JC, Colombo T, Long ME. Doxorubicin pharmacokinetics: Macromolecule binding, metabolism, and excretion in the context of a physiologic model. Journal of pharmaceutical sciences. 2002;91(6):1488-501. doi: 10.1002/jps.10161. PubMed PMID: 12115848.
17. Wood BJ, Poon RT, Locklin JK, Dreher MR, Ng KK, Eugeni M, et al. Phase I Study of Heat-Deployed Liposomal Doxorubicin during Radiofrequency Ablation for Hepatic Malignancies. Journal of Vascular and Interventional Radiology. 2012;23(2):248-55. PubMed Central PMCID: PMCA.
18. Hilmer SN, Cogger VC, Muller M, Le Couteur DG. The hepatic pharmacokinetics of doxorubicin and liposomal doxorubicin. Drug Metab Dispos. 2004;32(8):794-9. PubMed PMID: 15258103.
19. El-Kareh AW, Secomb TW. Two-mechanism peak concentration model for cellular pharmacodynamics of Doxorubicin. Neoplasia. 2005;7(7):705-13. PubMed PMID: 16026650; PubMed Central PMCID: PMC1501422.
20. Kerr DJ, Kerr AM, Freshney RI, Kaye SB. Comparative intracellular uptake of adriamycin and 4'-deoxydoxorubicin by non-small cell lung tumor cells in culture and its relationship to cell survival. Biochemical pharmacology. 1986;35(16):2817-23. PubMed PMID: 3741470.
21. Qian F, Stowe N, Liu EH, Saidel GM, Gao J. Quantification of in vivo doxorubicin transport from PLGA millirods in thermoablated rat livers. Journal of controlled release : official journal of the Controlled Release Society. 2003;91(1-2):157-66. PubMed PMID: 12932647; PubMed Central PMCID: PMCA.
22. Tofts PS, Brix G, Buckley DL, Evelhoch JL, Henderson E, Knopp MV, et al. Estimating kinetic parameters from dynamic contrast-enhanced T(1)-weighted MRI of a diffusable tracer: standardized quantities and symbols. J Magn Reson Imaging. 1999;10(3):223-32. Epub 1999/10/03. doi: 10.1002/(SICI)1522-2586(199909)10:3<223::AID-JMRI2>3.0.CO;2-S [pii]. PubMed PMID: 10508281.
23. Brizel DM, Klitzman B, Cook JM, Edwards J, Rosner G, Dewhirst MW. A comparison of tumor and normal tissue microvascular hematocrits and red cell fluxes in a rat window chamber model. Int J Radiat Oncol Biol Phys. 1993;25(2):269-76. Epub 1993/01/15. PubMed PMID: 8420874.
24. Gasselhuber A, Dreher MR, Rattay F, Wood BJ, Haemmerich D. Comparison of conventional chemotherapy, stealth liposomes and temperature-sensitive liposomes in a mathematical model. PloS one. 2012;7(10):e47453. doi: 10.1371/journal.pone.0047453. PubMed PMID: 23082168; PubMed Central PMCID: PMC3474827.

25. Poon RT, Borys N. Lyso-thermosensitive liposomal doxorubicin: a novel approach to enhance efficacy of thermal ablation of liver cancer. Expert Opin Pharmacother. 2009;10(2):333-43. Epub 2009/02/25. doi: 10.1517/14656560802677874. PubMed PMID: 19236203.
26. Brown RP, Delp MD, Lindstedt SL, Rhomberg LR, Beliles RP. Physiological parameter values for physiologically based pharmacokinetic models. Toxicol Ind Health. 1997;13(4):407-84. Epub 1997/07/01. PubMed PMID: 9249929.
27. Yuan F, Leunig M, Berk DA, Jain RK. Microvascular permeability of albumin, vascular surface area, and vascular volume measured in human adenocarcinoma LS174T using dorsal chamber in SCID mice. Microvasc Res. 1993;45(3):269-89. doi: 10.1006/mvre.1993.1024. PubMed PMID: 8321142.
28. Jain RK. Transport of molecules in the tumor interstitium: a review. Cancer Res. 1987;47(12):3039-51. Epub 1987/06/15. PubMed PMID: 3555767.
29. Swenson CE, Haemmerich D, Maul DH, Knox B, Ehrhart N, Reed RA. Increased Duration of Heating Boosts Local Drug Deposition during Radiofrequency Ablation in Combination with Thermally Sensitive Liposomes (ThermoDox) in a Porcine Model. PloS one. 2015;10(10):e0139752. doi: 10.1371/journal.pone.0139752. PubMed PMID: 26431204; PubMed Central PMCID: PMCPMC4592068.
30. Muggeo VM. Estimating regression models with unknown break-points. Stat Med. 2003;22(19):3055-71. doi: 10.1002/sim.1545. PubMed PMID: 12973787.
31. Team RC. R: A language and environment for statistical computing. R Foundation for Statistical Computing Vienna, Austria: R Foundation for Statistical Computing; 2016. Available from: https://www.R-project.org/.
